# Interferon regulatory factor 1 inactivation in human cancer

**DOI:** 10.1042/BSR20171672

**Published:** 2018-05-08

**Authors:** Khaldoon Alsamman, Omar S. El-Masry

**Affiliations:** Department of Clinical Laboratory Sciences, College of Applied Medical Sciences, Imam Abdulrahman Bin Faisal University, Dammam 31441, Saudi Arabia

**Keywords:** Cancer, Interferon Regulatory Factor, Oncogene, Tumor Suppressor

## Abstract

Interferon regulatory factors (IRFs) are a group of closely related proteins collectively referred to as the IRF family. Members of this family were originally recognized for their roles in inflammatory responses; however, recent research has suggested that they are also involved in tumor biology. This review focusses on current knowledge of the roles of IRF-1 and IRF-2 in human cancer, with particular attention paid to the impact of IRF-1 inactivation. The different mechanisms underlying IRF-1 inactivation and their implications for human cancers and the potential importance of IRF-1 in immunotherapy are also summarized.

## Introduction

In human cancers, the accumulation of genetic aberrations is known to affect the normal functions of several genes that control cell proliferation and survival. Amongst these genes is *IRF-1*, a member of the interferon regulatory factor (IRF) family. IRF-1 was first identified in 1988 as a transcription factor able to induce expression of the gene interferon β (*IFN-B*) [1]. The following year, IRF-2 was identified and found to suppress the function of IRF-1 [2]. Currently, the IRF family comprises ten members: IRF-1, IRF-2, IRF-3, IRF-4/Pip/ICSAT/LSIRF, IRF-5, IRF-6, IRF-7, IRF-8/interferon consensus sequence binding protein (ICSBP), IRF-9/ISGF3γ/p48, and IRF-10 [3–5]. IRF-1 to IRF-9 are present in mammals, but IRF-10 appears to be restricted to fish and chickens. As a result of the discovery of this extended group of IRFs, the terms ‘IRF kingdom’ and ‘IRF world’ have been coined [6].

**Figure 1:**
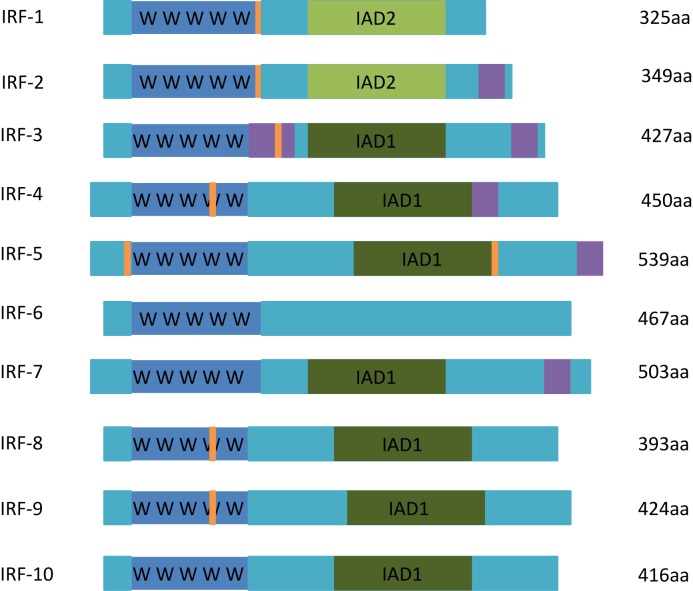
Illustration of the various functional domains of IRF family members All IRF family members contain a DBD (blue) and a regulatory domain (light blue). In addition, most IRFs possess a type 1 (dark green) or type 2 (light green) IAD. A repression domain that functions to repress gene expression (purple) is also present in some IRF family members. Finally, a nuclear localization signal domain (orange) is found in most IRFs.

All members of the IRF family exhibit significant homology in their N-terminal region, which contains a DNA-binding domain (DBD) that includes a cluster of five tryptophan residues. This DBD forms a helix-turn-helix motif and recognizes the interferon-stimulated response element (ISRE) in the promoters of genes targetted by IRFs [5,6]. The C-terminal region of most IRF family members is less conserved and contains an IRF-association domain (IAD) responsible for homomeric and heteromeric interactions with other proteins, including other IRF family members and non-IRF transcription factors and cofactors (e.g. PU.1 and E47) [3,4]. Two types of IAD have been identified, namely, IAD1 and IAD2. IAD1 is present in all members of the IRF family, with the exception of IRF-1 and IRF-2, in which IAD2 is found [3]. By mediating protein–protein interactions, these IADs confer specific roles and functions upon each member of the IRF family (Figure 1).

IRFs were originally recognized for their roles in innate and adaptive immunity, especially in the regulation of interferon-inducible genes in the interferon system [7]. However, recent studies have indicated their involvement in oncogenesis and other cellular responses.

IRF family members are playing a pivotal role in immune response. Amongst those, IRF-3 and IRF-7 whose antiviral role is well established. In addition, the hematopoietic factors, IRF-4 and 8. IRF-3 is expressed in all cell types and its expression is not triggered by viral infection or downstream to interferon [8]. Unlike IRF-3, IRF-7 expression is expressed downstream to interferon signaling pathway [9].

Expression of IRF-3 is up-regulated upon recognition of viral dsRNA by cellular receptor and, as a consequence of toll-like receptor-3 signaling, IRF-3 is phosphorylated and activated as a result of this post-translational modification [10]. This leads to formation of homo or heterodimers of IRF-3 and IRF-7, which translocate to nucleus and induce interferon-β, as well as, other interferon-stimulated genes (ISGs) after binding to cAMP-responsive element binding protein 1 (CREB) binding protein (CBP) [11].

Ubiquitination of IRF-3 targets its degradation by proteasome enzyme system, a process that can be triggered by propyl isomerase (Pin1) [12]. In contrast, ISG 15 (ISG-15); a ubiquitin-like protein can bind IRF-3, stabilizing it and increased its nuclear retention. This contributed to ISG-15 action in enhancing host antiviral response [13].

IRF-4 is expressed in B lymphocytes and dendritic cells and is thought to be required for B- and T-lymphocytes’ maturation and differentiation [14–16]. In this respect, an association between overexpression of IRF-4 and multiple myeloma was reported and was explained by translocation of this factor near to the locus of immunoglobulins [17]. Interestingly, IRF-4 is an endogenous antagonist of IRF-1, which is known for its tumor suppressing activity [18].

IRF-5 is another immunomodulatory factor, which has been recognized for its role as a regulator for type I interferon gene expression in response to viral infections. It has a role in regulation and development of host immune response and autoimmune responses; hence it has been recognized as a susceptibility gene for autoimmune disorders [19]. The functions of IRF-5 are extended to other disease categories including cancer, obesity, pain mediation [20], and cardiovascular disease [21]. The pleiotropic nature of IRF-5 functions could be also confirmed by its reported role as a regulator of cell growth and apoptosis, which explains its potential as tumor suppressor being down-regulated in malignant tissues [22]. In addition, metabolic activities of IRF-5 have been also reported [23].

IRF-6 is a unique member of IRF family being the only family member to be essential for embryogenesis [24]. IRF-6 is also a crucial protein for proliferation and differentiation of keratinocytes, which makes it an important element to the process of wound healing (reviewed in [25]). Defects in *IRF-6* gene have been observed in patients with cleft lip and/or palate. In addition, aberrations in *IRF-6* predisposes for squamous cell carcinoma and defective development of mammary gland [26]. Adding to this information, recently, a novel role of IRF-6 was reported implicating IRF-6 in development of exocrine glands as another function besides its role as a tumor suppressor [27].

Human *IRF-7* gene can be induced by type I interferon and tumor necrosis factor α (TNF-α). On the other hand, regulation of type I interferon gene expression by IRF-7 has been reported, hence the relation between IRF-7 and type I interferon could be described as mutual [9,28]. This was confirmed by the finding that homozygous deletion of IRF-7 in an animal model abolished expression of type I interferon-regulated genes following activation of TLR-9 or viral infections [29]. Activation of IRF-7 is also phosphorylation dependent and is an outcome of TLR-3, -7, -8 and -9 signaling pathways [30].

IRF-8, also known as ICSBP is expressed solely in lymphoid and myeloid progenitors [31]. The function of this member depends on its interaction with other IRF members including IRF-1 and 4 [32]. IRF-1–IRF-8 heterodimer suppresses ISG-15, whereas ISG-15 is induced by IRF-4–IRF-8 complex [33]. Additionally, macrophages differentiation and activation during inflammatory response is also activated by IRF-1–IRF-8 heterodimer [34].

IRF-9, p48, or ISGF3-γ contributes to the antiviral response of interferon α, β, and γ. This role is achieved primarily by the binding of IRF-9 to interferon stimulated gene factor3, which interacts with ISRE and regulates ISGs [35,36].

This review discusses the functions of IRF-1 and IRF-2 in human cancers, with a focus on the potential contribution of IRF-1 inactivation to human carcinogenesis and the future of IRF-1 as a therapeutic target.

## Antioncogenic and oncogenic potential of IRF-1 and IRF-2

The role of the IRF family in oncogenesis was first noted in 1993, when overexpression of IRF-2 was found to transform NIH 3T3 cells and enhance their tumorigenicity in nude mice, a phenotype that was shown to be reversed by IRF-1 overexpression [37]. An antioncogenic function for IRF-1 was also implied by the finding that overexpression of the Ha-*ras* oncogene was seen to result in transformation of *IRF-1*^−/−^ but not wild-type mouse embryonic fibroblasts (MEFs) [38]. Surprisingly, ectopic expression of the *N-ras* oncogene in some myeloid cell lines has been shown to suppress proliferation and up-regulate the cyclin-dependent kinase (CDK) inhibitor p21^WAF1/CIP1^. This suppression was found to be associated with up-regulation of IRF-1, further reinforcing the notion that this IRF exerts an antioncogenic effect [39]. Moreover, overexpression of IRF-1 in a wide range of different cell types from humans, mice, and even hamsters has been reported to cause growth inhibition [40–43]. In contrast with other tumor suppressors, loss of IRF-1 function rarely induces oncogenicity; however, IRF-1 inactivation is a cofactor in increased risk of tumorigenesis mediated by p53 nullizygosity or Ha-*ras* oncogene overexpression [44].

The antiproliferative effect of IRF-1 has chiefly been attributed to its induction of the expression of certain target genes that down-regulate cell growth. These genes include protein kinase R (*PKR*) and signal transducer and activator of transcription (STAT) 1 (*STAT1*) in the Janus kinase (JAK)-STAT pathway [45,46]. The panel of IRF-1-induced genes also includes those encoding caspases, tumor necrosis factor-related apoptosis-inducing ligand (*TRAIL*), and lysyl oxidase (*LOX*), the latter of which has also been identified as a tumor suppressor [47]. Caspases are known for their protease activity and their activation and participation in the apoptotic cascade. IRF-1 mediates IFN-γ-induced apoptosis in ovarian cancer cell lines via induction of caspase-1 expression [48], and up-regulates caspase-8 expression in response to IFN-γ/STAT1 signaling as part of a mechanism that sensitizes cells to apoptosis [49]. IRF-1 also binds to unique sites in the p53 up-regulated modulator of apoptosis (*PUMA*) promoter, resulting in up-regulation of the intrinsic apoptosis pathway [50]. Cyclin D1 and survivin have also been reported as downstream targets of IRF-1, and *in vitro* activation of this IRF decreases cyclin D1 expression and CDK 4 (CDK4) activity [51].

The inhibitor of apoptosis, survivin, is a potential target for cancer therapy as its overexpression by tumor cells promotes their survival. Notably, overexpression of IRF-1 in breast carcinoma cells has been found to result in a 15-fold down-regulation of survivin protein levels [52], which has been attributed to the suppression of cyclin B1, CDK-1, cyclin E, E2F1, CDK2, and CDK4 expression [53]. However, survivin may also be regulated in human cancer cells by other IRF-1 signaling pathways or directly by IRF-1 itself [53]. IRF-1 also induces p21-mediated G_1_ cell cycle arrest in such cells [53]. IRF-1 is believed to prevent oncogenesis through initiation of apoptosis, as demonstrated by the IRF-1- and p53-mediated apoptosis, but not cell cycle arrest, of Ha-*ras*-overexpressing wild-type MEFs following treatment with ionizing radiation or anticancer drugs [5,6].

IRF-1 and p53, both of which are tumor suppressors, regulate DNA damage-induced apoptosis independently or co-operatively. For example, DNA damage-induced apoptosis of mature T lymphocytes relies solely on IRF-1 [54], whereas that of thymocytes depends on p53 [54,55]; thus, cell type and differentiation stage dictate the tumor suppressor that regulates this process. Furthermore, a role for IRF-1 in the DNA damage repair mechanism has been described. By using ChIP coupled to a CpG island microarray, Frontini and colleagues [56] identified 202 IRF-1 binding sites in the human genome, leading to the discovery that BRCA1-interacting protein C-terminal helicase 1 (BRIP1, also known as Fanconi anemia gene J) is up-regulated as a result of IFN-mediated induction of IRF-1 expression. BRIP1 has been implicated in breast cancer susceptibility and is known for its DNA repair function [57]. Its association with IRF-1 further supports the potential involvement of this regulatory protein in antioncogenic activity.

The concept that *IRF-1* has an antioncogenic effect via cell cycle regulation is supported by change in its expression throughout the cell cycle [37]. Such changes inhibit the growth of cells with damaged DNA by inducing G_1_ cell cycle arrest, an effect that is dependent on ataxia telangiectasia mutated (ATM) and mediated by binding of the promoter region of p21^WAF1/CIP1^, which contains binding sites for both IRF-1 and p53. It has also been reported that activation of IRF-1 results in the expression of genes directly involved in various other cellular processes, including regulation of the T cell-mediated immune response to viral infection. Deletion or mutation of *IRF-1* and exon skipping (a form of RNA splicing to skip faulty exons) in the corresponding mRNA are also associated with the development of various hematopoietic malignancies and syndromes [58].

In contrast with IRF-1, IRF-2 exerts a pro-oncogenic effect. IRF-2 has been reported to be up-regulated in pancreatic cancer, in which it is associated with tumor size and differentiation, tumor node metastasis stage, and survival [59]. Moreover, overexpression of the *IRF-2* gene has been shown to prevent *N-ras*-induced growth suppression, confirming its pro-oncogenic role [60]. One study has attributed the oncogenic activity of IRF-2 to its ability to bind to the ISRE via its DNA-binding/transcription repression domain, preventing IRF-1 and other IRF family members from binding to the same DNA response element [61]. In addition, IRF-2 regulates transcription of downstream targets involved in oncogenesis, such as histone H4 [62,63]. A further mechanism underlying the oncogenic activity of IRF-2 involves its interaction with murine double minute-2 (MDM2), an enzyme that catalyzes p53 ubiquitination and degradation via the proteasome pathway [64]. Target genes of IRF-1 and IRF-2 are summarized in Table 1.

**Table 1:** Target genes of IRF-1 and IRF-2

IRF-1 Targets	Action	IRF-2 targets	Action
**Cell cycle regulatory genes**		**DNA-binding activity**	
CDK inhibitor p21WAF1/CIP1 [65]	Up-regulation	ISRE [61]	Inhibition of IRF-1 transacting activity
Cyclin D1 [51]	Down-regulation	H4 [63]	Regulation of gene expression
Cyclin E [65]	Down-regulation	**Anti-apoptotic**	
CDK2 [65]	Down-regulation	*MDM2* [64]	Interaction and inhibition of p53-mediated effects
CDK4 [65]	Down-regulation		
E2F [65]	Down-regulation		
**Growth suppression genes**			
PKR [46]	Up-regulation		
LOX [47]	Up-regulation		
**Apoptosis-inducing genes**			
*Caspase 1* [48]	Activation		
Caspase 3 [66]	Activation		
Caspase 7 [66]	Activation		
Caspase 8 [66]	Activation		
*Caspase 8* [49]	Activation		
*PUMA* [50]	Up-regulation		
*BRIP1 (Fanconi Anemia gene J)* [56]	Up-regulation		
*TRAIL* [67]	Up-regulation		
**Immunomodulation**			
*MHC-I* [68]	Up-regulation		
*ISG-15* [69]	Up-regulation		

Gene names are italicized.

**Figure 2:**
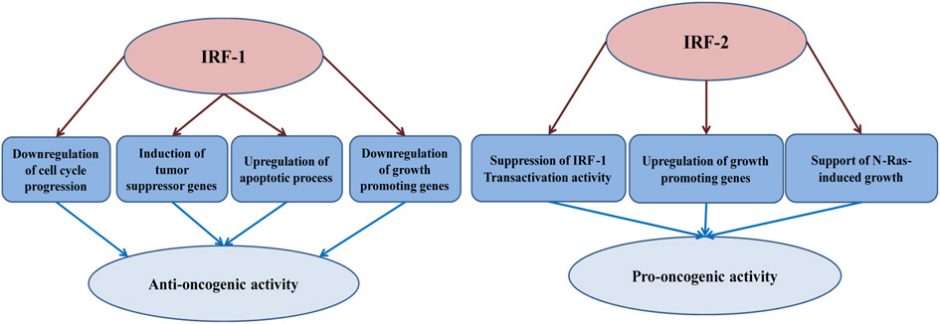
The anti- and pro-oncogenic activity of IRF-1 and IRF-2 IRF-1 anti-oncogenic activity is attributed to four main mechanisms: (I) Derailing of cell cycle; (II) Down-regulation of growth promoting genes; (III) Induction of tumor suppressor genes, and (IV) Up-regulation of apoptotic machinery. The pro-oncogenic potential of IRF-2 is owed to: (i) Suppression of IRF-1 activity; (ii) up-regulation of growth promoting genes, and (iii) supporting oncogenes-induced growth.

In conclusion, IRF-1 operates as a tumor suppressor whose loss, in combination with other genetic alterations, may significantly increase risk of malignancy. In the following sections, we summarize its role in different human cancers and the range of mechanisms by which this tumor suppressor loses its function (Figure 2 summarizes the anti- and pro-oncogenic potential of IRF-1 and 2).

## IRF-1 in human cancers

### Human leukemia and pre-leukemic myelodysplasia

Human leukemia and pre-leukemic myelodysplastic syndrome (MDS) are characterized by a remarkable cytogenetic abnormality, namely, the loss of chromosome 5 or a deletion within its long arm (del(5q) or 5q−) [70]. This aberration accounts for 30% of MDS cases, 15% of *de novo* acute myelogenous leukemia (AML) cases, 50% of cases of secondary AML arising from MDS, and 2% of *de novo* acute lymphocytic leukemia cases [71,72]. Willman and colleagues [70] proposed that a tumor suppressor gene must be located in the common deleted segment (5q31), and eventually succeeded in mapping *IRF-1* to band 31.1 of chromosome 5 using fluorescence *in situ* hybridization with a 19-kb *IRF-1* probe. This probe only hybridized to sequences on chromosome 5q and was precisely mapped to 5q31.1 by computer-assisted fluorescence microscopy. Once the *IRF-1* locus had been determined, a full-length *IRF-1* cDNA probe was used to perform Southern blotting of DNA from patients with different types of leukemia and MDS associated with del(5q) to confirm IRF-1 inactivation in clinical samples. The results of the present study indicated an unusual instability in the 5q region, as deletion of one *IRF-1* allele was accompanied by rearrangement or deletion of the second allele in some cases. This led to the conclusion that deletions or rearrangements are more frequent than point mutations at this locus in human leukemia and MDS, and loss of *IRF-1* may be critical in the development of AML and MDS [70].

Another group has also investigated the proposal that *IRF-1* loss is key in 5q− syndrome development, finding that 85.7 % of the patients included in their study exhibited loss of one allele of the *IRF-1* gene, with no evidence of homozygous loss [73]. This finding is consistent with a detailed examination of the ‘critical region’ on 5q affected in MDS and AML patients, which contains crucial genes with tumor suppressing potential, such as interleukin 3 (*IL-3*), *IL-4*, *IL-5*, and *CSF2* [74], which *IRF-1* maps closely [70].

In the same context, Harada and colleagues [75] reported that a significant proportion of *IRF-1* mRNA transcripts obtained from the bone marrow and peripheral mononuclear cells of patients with MDS or leukemia secondary to MDS lacks exon 2 (containing the initiation codon) and exon 3 as a result of accelerated exon skipping. The resulting IRF-1 protein lacks the ability to bind DNA and its tumor-suppressing activity is consequently lost. Thus, accelerated exon skipping comprises a second mechanism, in addition to *IRF-1* loss as a result of DNA damage, by which *IRF-1* is inactivated. This process may explain the development of hematopoietic malignancies in some 5q− syndrome cases in which both copies of the *IRF-1* gene are retained. To validate this proposal, Green and colleagues [76] developed quantitative competitive RT-PCR assays to measure levels of full-length and exon-skipped IRF-1 transcripts (IRF-1∆2 and IRF-1∆2,3) in acute promyelocytic leukemia (APL), AML, and MDS patients, with a particular focus on those carrying an *IRF-1* allele deletion. Their results showed that accelerated exon skipping is common in patients with a 5q deletion and one deleted *IRF-1* allele, and occurs in most APL cases (in which IRF-1 protein expression was found to be absent). Such exon skipping thus leads to loss of IRF-1 function and increases risk of malignancy [76].

To evaluate the extent to which exon skipping-induced IRF-1 inactivation is involved in oncogenesis, investigation of the association between exon-skipped IRF-1 transcripts and other forms of leukemia was also necessary. Mutational analysis and studies of IRF-1 expression patterns have revealed a four-fold reduction in levels of full-length *IRF-1* mRNA and elevated presence of abnormal splice variants in chronic myeloid leukemia (CML) patients [77]. This lends weight to the idea that production of non-functional splice variants is another mechanism underlying IRF-1 inactivation, and one that is particularly relevant to CML. Unlike the IRF-1 splice variants observed in AML, those in CML lack exons 7, 8, and 9, in addition to the AUG initiation codon in exon 2 and the DBD [77]. Loss of exons 7, 8, and 9 compromises the ability of IRF-1 to heterodimerize with cofactors and/or other IRF members, such as IRF-8, levels of which have also been reported to be reduced in CML patients [78].

Examining leukemogenesis from a different perspective, Preisler and colleagues [79] compared the *IRF-1*/*IRF-2* gene expression ratio in AML and normal marrow, concluding that the balance between these factors, rather than the expression level of either in isolation, ultimately determines phenotype. Their study revealed that this ratio was significantly lower in AML patients as a result of low IRF-1 and high IRF-2 transcript levels, and indicated that IRF-1-responsive genes reduce AML risk. In contrast, malignant transformation related to stimulation of *IRF-2* gene appears to be relatively common in leukemogenesis [79].

### Human breast cancer

Following the discovery of a role for IRF-1 in human leukemia and pre-leukemic myelodysplasia, researchers began to question whether it and other IRF family members might be similarly implicated in other cancers, particularly solid tumors. In one retrospective study, IRF-1 but not IRF-2 was found to be expressed in normal breast tissue, whereas levels of the former were shown to be lower and those of the latter higher in high-grade ductal carcinoma *in situ* (DCIS) and lymph node-positive invasive ductal cancer [80]. This finding suggests that IRF-1 and IRF-2 protein expression profiles are altered in human breast cancer, consistent with their respective proposed roles as a tumor suppressor and oncoprotein.

In the development of previous work, Connett and colleagues [81] used immunohistochemical tissue microarrays on a larger sample of invasive breast carcinoma specimens to show that neoplastic breast tissues are less likely to maintain IRF-1 expression than adjacent normal tissues. They also demonstrated that IRF-1 expression negatively correlates with tumor size, confirming that loss of IRF-1 is associated with breast carcinogenesis [81]. No correlation was noted between IRF-2 and clinical parameters in this study; however, this may be explained at least in part by the complex nature of the regulatory relationship between IRF-1 and IRF-2 in their exertion of tumor suppressive and oncogenic effects, respectively [80,81]. Moreover, the mechanism by which dysregulation of IRF-1 and IRF-2 affects breast carcinoma cells has been proposed to involve disruption of the IRF-1/IRF-2 ratio [82], and as this ratio has been reported to change during the cell cycle, monitoring the status of IRF-1 and IRF-2 in such histological studies is highly challenging [43]. Other studies have suggested that post-translational modifications of IRF-1 and IRF-2 are more critical to the activity of these proteins than the levels at which they are present [83–85]. These observations indicate that further molecular investigations are required to establish an association between IRF-1 and IRF-2 and breast tumorigenesis.

At the genetic level, there have been no reports of point mutations that cause IRF-1 inactivation in breast cancer; however, an *IRF-1* polymorphism (A4396G) has been identified in breast cancer cell lines, and has been found to be more frequent amongst African Americans [86]. It is unknown whether this variant contributes to breast oncogenesis. The nucleotide substitution involved does not change the sequence of the translated protein, and there is no evidence that this polymorphism results in the introduction of a new active splicing site, which can lead to IRF-1 inactivation in human cancers [86]. It has been hypothesized that this variant influences the binding of certain critical transcription factors with tumor-suppressing potential, including microphthalmia transcription factor (MITF), which activates INK4A, a tumor suppressor that inhibits cell cycle progression [86,87]. The identification of this polymorphism highlights the need for more thorough investigation of genetic changes in *IRF-1* in patients with breast carcinoma and other tumors.

A lack of sufficient evidence concerning the exact contribution of IRF-1 to breast carcinogenesis, together with the well-documented role of this protein in hematopoietic malignancies, triggered a number of comparative genomic hybridization studies. As a result, loss of the 5q31.1 region, to which IRF-1 has been mapped, was found to be common in breast tumors [88]. In addition, 5q12-31 deletions were noted in 11% of sporadic breast cancers, and 5q31.1 loss was observed in 50% of *BRCA1* mutation-positive breast tumors. Given that somatic loss of IRF-1 may be a critical event in breast oncogenesis, in 2010, Cavalli and colleagues [88] investigated its incidence in 52 patients with invasive breast tumors. Loss of heterozygosity (LOH) at the *IRF-1* locus was found in 32% of cases, providing evidence of a tumor-suppressive effect of IRF-1 in breast cancer [88].

### Other human cancers

The establishment of a tumor-suppressing role for IRF-1 in breast cancer and leukemia laid the foundations for further investigations exploring its function in other cancers. One such study revealed that 50% of gastric tumors exhibit LOH at the 5q region implying a critical contribution of IRF-1 to the development of stomach carcinoma [89]. In another investigation, 5q31.1 was reported to be lost in primary esophageal carcinoma, and was the smallest commonly deleted region in 57% of the specimens tested [90], implicating IRF-1 in the pathogenesis of this malignancy.

For the correct interpretation of these findings, it was important to test the so-called ‘two-hit hypothesis’ in relation to *IRF-1* to confirm the role of this gene in esophageal carcinoma and stomach adenocarcinoma [91]. This hypothesis proposes that loss of function of a critical tumor suppressor gene requires allelic changes in both sister chromatids of the chromosome concerned. Sequencing of the *IRF-1* gene in gastric adenocarcinoma tissues confirmed LOH at this locus, and led to the identification of a loss-of-function point mutation resulting in a methionine-to-leucine substitution at codon 8 [38]. This mutation attenuates the transcriptional activity of *IRF-1* and consequently, its tumor-suppressing capability is lost. Although it is not clear how this mutation brings about this effect, it has been proposed that it may enhance the interaction of IRF-1 with other factors, impairing the function of this protein as a regulator of transcription [38]. Wang and colleagues [92] have further investigated the part played by IRFs in esophageal malignancies by measuring patterns of IRF-1 and IRF-2 protein expression in esophageal squamous cell carcinoma (ESCC) and correlating them with the clinical features of this disease. They found expression of IRF-1 to be decreased and that of IRF-2 increased in ESCCs compared with matched normal esophageal tissue. These results demonstrate that IRF-2 expression may be positively correlated with the progression of this cancer, adding to the multitude of observations supporting opposite roles for IRF-1 and IRF-2 in tumorigenesis.

A separate group has reported a correlation between IRF-1 expression in human melanoma tissue specimens and less advanced disease, although they were not able to demonstrate a clear relationship between IRF-2 expression and this malignancy in this work [93]. In an examination of cervical cancer tissues, Lee and colleagues [94] noted the presence of five different splice variants of *IRF-1* mRNA lacking particular combinations of exons 7, 8, and 9. This alternative splicing results in the absence of the IRF-1 functional domain or the generation of a truncated protein with aberrant transcriptional activity that interferes with that of wild-type IRF-1. Thus, alternative splicing affecting exons 7, 8, and 9 may be another critical mechanism negatively regulating IRF-1 in cervical cancer.

Recently, it has also been reported that high IRF-1 expression in hepatocellular carcinoma (HCC) is associated with better outcome in terms of frequency of recurrence following surgical resection. In contrast, overexpression of IRF-2 is associated with increased probability of recurrence [95]. In addition, a high IRF-2/IRF-1 protein ratio positively correlates with tumor invasion and metastatic ability in human HCC cell lines [95]. The involvement of IRF-1 and IRF-2 in the progression of human pancreatic cancer has also been reported [96]. Whereas *IRF-1* expression is reduced in pancreatic cancer specimens compared with adjacent normal tissues, *IRF-2* gene expression is up-regulated. In the same work, it was also found that up-regulation of IRF-1, but not IRF-2, leads to better tumor differentiation, enhanced lymphocyte infiltration, smaller tumor mass, and longer survival [96]. Subsequent research using an *in vitro* model of pancreatic cancer has confirmed these clinical observations and thus, the tumor-suppressing and -promoting potentials of IRF-1 and IRF-2, respectively [96].

Kuroboshi and colleagues [97] have expressed doubt concerning the nature of altered IRF-1 expression in uterine endometrial carcinoma compared with pre- and postmenopausal endometrial tissue, suggesting that the modified levels of this protein could be either an outcome or a cause of the development of this malignancy. Nevertheless, considered together with other findings, whereas *IRF-2* may be classified as an oncogene, these results are consistent with *IRF-1* having tumor-suppressing potential. Table 2 shows a timeline of the first recognition of the influence of IRF-1 in different human cancers.

**Table 2:** Timeline represent the year of IRF-1 first involvement in different cancer types

Cancer type	Year first reported	References
***Leukemia and pre-leukemia myelodysplasia***	**1993**	[70,73,76,77,79]
***Stomach carcinoma***	**1996**	[89]
***Esophageal carcinoma***	**1996**	[90,92]
***Gastric adenocarcinoma***	**1998**	[38]
***Breast cancer***	**1998**	[80,81,86,88,98]
***Skin melanoma***	**1999**	[93]
***Uterine endometrial carcinoma***	**2003**	[97]
***Cervical cancer***	**2006**	[94]
***HCC***	**2013**	[95]
***Pancreatic cancer***	**2014**	[96]

## Inactivation of IRF-1 in human cancers

Several mechanisms responsible for attenuated IRF-1 transcriptional activity in human malignancies have been reported. The following section describes the different routes by which the function of IRF-1 and, thereby, its tumor-suppressing role may be lost in human cancers.

### Genetic modulation of IRF-1 activity

Alterations in the *IRF-1* gene have been reported in both hematologic malignancies and solid tumors. For instance, inactivating rearrangements or deletions in *IRF-1* have been reported in AML [70], and LOH of this gene has been observed in gastric and esopharyngeal cancers and renal cell carcinoma [99]. In addition, a missense mutation in exon 2 of *IRF-1* has been identified in stomach cancer. This alteration was accompanied by loss of IRF-1 transcriptional and, consequently, tumor-suppressing activity [38]. We previously mentioned in this review that genetic alterations in *IRF-1* have also been documented in breast cancer [88]. Green and colleagues [76] have also described a single *IRF-1* allele deletion in AML and MDS patients.

### Transcriptional modulation of IRF-1 activity

*IRF-1* mRNA is subject to several alterations that ultimately lead to loss of function. For example, Lee and colleagues [94] have identified splice variants of *IRF-1* mRNA missing combinations of exons 7, 8, and 9. These variants are highly expressed in cervical cancer tissue and associated with attenuated IRF-1 transcriptional activity. *miR-23a*, which binds to the 3′-UTR of *IRF-1* mRNA, is overexpressed in gastric adenocarcinoma, resulting in IRF-1 down-regulation and loss of transcriptional activity, in turn enhancing pro-proliferative and anti-apoptotic conditions [100]. Skipped exons in IRF-1 transcripts have also been reported. Skipping of exon 2 in mutant *IRF-1* is associated with an absent DBD and loss of the tumor-suppressing action of the encoded protein [101]. Moreover, faulty *IRF-1* mRNA can result from accelerated exon skipping, with affected transcripts lacking a translation initiation site. This constitutes one of the mechanisms leading to IRF-1 inactivation in hematopoietic cancers [76].

### Proteomic modulation of IRF-1 activity

#### SUMOylation

SUMOylation is a post-translational modification in which lysine residues are modified by attachment of a SUMO group [102]. SUMOylation of IRF-1 stabilizes this protein and protects it from degradation, but also leads to loss of its transcriptional activity, inhibiting IRF-1-mediated apoptosis and tumor-suppressing activity; therefore, levels of IRF-1 SUMOylation are increased in tumor cells [103]. Indeed, SUMO-IRF-1 induces transformation of NIH 3T3 cells in a dose-dependent manner, implying that following SUMOylation, IRF-1 loses its antioncogenic activity and mimics the oncogenic factor IRF-2 [104].

#### Oncoproteins

Viruses have evolved various strategies to survive immune responses. Human papillomavirus (HPV), which is associated with increased risk of cervical cancer [105], counteracts interferon signaling to evade the human immune defense system. It has been reported that HPV oncoproteins can bind and inhibit the transcriptional activity of interferon-regulatory proteins including IRF-1 [106]. For example, HPV E6 inhibits IRF-3 activity, and HPV E7 binds to and inactivates IRF-1 and IRF-9 [107]. An early report from Park and colleagues [108] indicated a physical interaction between HPV E7 and IRF-1 leading to loss of transcriptional activity, thought to be mediated by histone deacetylation, and abrogation of IFN-α signaling.

#### Nucleophosmin

Nucleophosmin (NPM) predominantly acts as a nuclear shuttling protein; however, translocation of the *NPM* gene is associated with mislocalization of this protein to the cytoplasm, which has been implicated in loss of the function and antioncogenic effects of IRF-1. Cytosolic NPM has been observed in clinical AML specimens [109]. Moreover, overexpression of this protein has been noted in leukemia cell lines, and high levels correlate with transformation of NIH 3T3 cells [110]. Anti-apoptotic proteins of the GAGE family (a group of highly related tumor antigens) can bind and stabilize NPM/B23, which is indirectly involved in loss of IRF-1 function. In addition, interaction between GAGE proteins and IRF-1 has been reported in cancer cells, possibly explaining GAGE-induced cell survival and resistance to IFN-γ treatment [111].

## Therapeutic targetting of IRF-1 in human cancers

**Figure 3:**
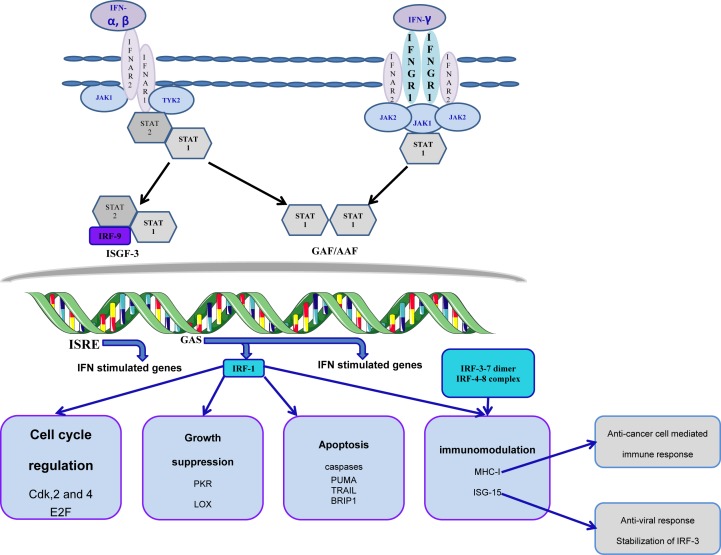
Signal transduction pathway and cross-talk in the interferon system Stimulation of type I and II interferon receptor recruits Janus and tyrosine kinases that phosphorylates and activates STAT. STAT proteins trigger nuclear translocation of IRF members where they interact with ISRE to regulate expression of ISG resulting in various physiological responses.

Cancer models have provided evidence on the antitumor potential of interferon and importance of signaling through type I interferon receptor in mediation of anticancer immune response [112]. Importantly, *IRF-1* is induced downstream to IFN-γ-type I interferon receptor signal transduction cascade (Figure 3). In turn, IRF-1 contributes to modulation of immune response besides suppression of cell growth and transformation. Amongst targets of *IRF-1* genes is the major histocompatibility class I (*MHCI*) gene. Induction of MHCI contributes to the long-known antitumor potential of IRF-1 enhancing tumor antigen presentation that improves the efficacy of the cell-mediated antitumor immune response. IFN-γ also triggers clonal expansion and activation of T cells and production of MHCII [113]. Therefore, functional type I IFN signaling and local expression of IFN-stimulated genes by tumors are cofactors for better disease prognosis and outcomes [114].

That IRF-1 is a potential target for new therapies has been highlighted by correlations between its inactivation and several types of human cancers. However, the exact IRF-1-related mechanisms that might be targetted to benefit therapy of human malignancies are not yet fully clear. It has been reported that an abnormally low IRF-1/IRF-2 ratio due to defective transcription is a basic characteristic of leukemogenesis, but that administration of certain cytokines (e.g. IL-4) can return this ratio to normal levels [79]. Similarly, Yoshino and colleagues [115] have suggested that this ratio has prognostic value with respect to diffusely infiltrating astrocytomas, and consider both IRFs to represent targets of future therapies. Consistent with this, it has been proposed that increasing the IRF-1/IRF-2 ratio may serve as a novel therapeutic strategy for pancreatic cancer [96]. Interestingly, a recent study has pointed out that down-regulation of IRF-1 may overcome resistance to anti-angiogenic drugs in glioblastoma [116]. There is also evidence that IRF-1 regulates both second mitochondria-derived activator of caspases (SMAC) and the pro-inflammatory response, suggesting that its up-regulation in human cancers could favor apoptosis [117]. With respect to breast cancer, IRF-1 has been identified as an effector that restores the sensitivity of estrogen receptor-positive metastatic breast carcinoma to the anti-estrogenic drug fulvestrant [118].

A recent study [119] has implicated the inactivation of type I interferon receptor chain (IFNAR1) in colorectal cancer (CRC) development and patients’ overall poor prognosis. Authors have reported that genetic or pharmacological stabilization of IFNAR1 can improve CRC patients’ response to chimeric antigen receptor treatment and inhibition of programmed cell death protein 1 (PD-1), which, in turn increases the efficacy of tumor immunotherapy by augmenting the activity and the number of cytotoxic T cells in the tumor niche [120]. Down-regulation of IFN signaling is associated with reduced expression of IFN-induced genes; this is indicated by lower nuclear levels of p-STAT2 in malignant colorectal tissues as compared with normal tissue specimens [119]. It has been reported that colorectal tumor microenvironment associated stress (hypoxia, for example) results in production of lower levels of IFNAR1 in CRC models [121]. In this context, significant higher levels of IFNAR1 were observed in colorectal normal tissues when compared with CRC tissues [119].

## Conclusion

Up-regulation of IRF-1 in cancerous lesions is regarded as an approach that could improve prognosis and alleviate resistance to immunotherapy [95,122]. In addition, measuring IRF-1 expression within malignancies is considerably helpful in determining prognosis and predicting response to immunotherapy. The various mechanisms involved in IRF-1 inactivation in human cancers involve processes at DNA, RNA, and protein levels. Concerning DNA, LOH along with monosomy and mutation play a major role in limiting the functionality of IRF-1, ultimately resulting in oncogenesis. From the transcriptional perspective, exon skipping and alternative splicing are the two key events leading to reduced IRF-1 activity. Finally, SUMOylation, oncoproteins, and NPM have a significant impact on the IRF-1 protein and oncogenesis (Table 3). The data summarized in this review suggest that more research should be conducted to explore the potential utility of the IRF-1 as a therapeutic target in cancer.

**Table 3:** Mechanisms involved in IRF-1 inactivation at various molecular levels.

Level	Inactivation mechanisms		
***DNA***	LOH	Monosomy	Mutations
***mRNA***	Exon skipping	Faulty splicing	
***Protein***	SUMOylation	Oncoprotein	

## Abbreviations

AML: acute myelogenous leukemia
APL: acute promyelocytic leukemia
BRCA1: breast cancer susceptibility gene 1
BRIP1: BRCA1-interacting protein C-terminal helicase 1
CDK: cyclin-dependent kinase
CML: chronic myeloid leukemia
CRC: colorectal cancer
DBD: DNA-binding domain
ESCC: esophageal squamous cell carcinoma
HCC: hepatocellular carcinoma
HPV: human papillomavirus
IAD: IRF-association domain
ICSBP: interferon consensus sequence binding protein
IFN: interferon
IFNAR1: type I interferon receptor chain
IRF-1: interferon regulatory factor 1
ISG: interferon-stimulated gene
ISGF-3: interferon-stimulated gene factor 3
ISRE: interferon-stimulated response element
LOH: loss of heterozygosity
MEF: mouse embryonic fibroblast
MDS: myelodysplastic syndrome
MHCI: major histocompatibility class I
NPM: nucleophosmin
STAT: signal transducer and activator of transcription
SUMO: small ubiquitin-like modifier
